# Temporal Patterns in Fruit and Vegetable Intake Among Racially and Ethnically Diverse Children and Adolescents in California

**DOI:** 10.5888/pcd21.230162

**Published:** 2024-02-08

**Authors:** Maria Elena Acosta, Emma V. Sanchez-Vaznaugh, Mika Matsuzaki, Nancy Barba, Brisa N. Sánchez

**Affiliations:** 1 Department of Public Health, San Francisco State University, San Francisco, California; 2 Department of International Health, Bloomberg School of Public Health, John Hopkins University, Baltimore, Maryland; 3 Department of Epidemiology and Biostatistics, Drexel University School of Public Health, Philadelphia, Pennsylvania

## Abstract

**Introduction:**

Childhood dietary behaviors, including fruit and vegetable intake, are associated with adult health. Most children do not meet daily recommended servings of fruits and vegetables. Less is known about temporal patterns in fruit and vegetable consumption or if they vary by race and ethnicity. We investigated temporal patterns in fruit and vegetable intake among California school-age children and adolescents overall and by race and ethnicity.

**Methods:**

We used 2-year cross-sectional datasets from the child and adolescent samples in the California Health Interview Surveys from 2011–2012 through 2019–2020 and modified Poisson regression models to estimate the likelihood of consuming 5 or more servings of fruits and vegetables in 2013–2016 and 2017–2020 compared with 2011–2012. Models controlled for age, race and ethnicity, gender, citizenship status, family income, and adult education and tested for differences by race and ethnicity. The samples included 16,125 children aged 5 to 11 years and 9,672 adolescents aged 12 to 17 years.

**Results:**

Overall, 29.3% of children and 25.9% of adolescents reported intake of 5 or more fruits and vegetables per day. Among children, adjusted prevalence ratios (PR) of fruit and vegetable intake were higher in 2013–2016 (PR,1.25; 95% CI, 1.11–1.42) and 2017–2020 (PR,1.13; 95% CI, 0.99–1.30) compared with 2011–2012. Among adolescents, the adjusted prevalence did not differ significantly over time. We found no evidence of differential associations by race and ethnicity for children and adolescents.

**Conclusion:**

We found favorable temporal changes in fruit and vegetable consumption among children, but not among adolescents. Monitoring temporal patterns in fruit and vegetable intake remains critical for planning population-level interventions to increase consumption.

**Summary**

**What is already known on this topic?**

Many children do not meet US Department of Agriculture dietary guidelines, which call for 5 or more servings of fruits and vegetables a day.

**What is added by this report?**

We used racially and ethnically diverse sample data for California children and adolescents from 2011 through 2020 waves of the California Health Interview Survey. Among children, but not adolescents, fruit and vegetable intake was higher in the 2013–2016 and 2017–2020 time periods compared with 2011–2012. We saw no evidence of temporal differences in intake by race or ethnicity.

**What are the implications for public health practice?**

Fruit and vegetable intake is important for diet quality and future health outcomes. To improve population health, continued efforts are needed to promote fruit and vegetable intake.

## Introduction

Poor diet and related behaviors in childhood may be a risk factor for chronic diseases later in life (1). The average Healthy Eating Index–2015 score in 2015–2016 among US children was 55 and among adolescents, 51, suggesting overall poor diet quality (2). Fruits and vegetables are especially important for maintaining health and preventing chronic diseases because they provide essential nutrients, such as vitamins and fiber (3). The 2020–2025 Dietary Guidelines for Americans recommend children and adolescents consume daily servings of fruits (1–2.5 cups equivalence) and vegetables (1–4 cups equivalence), depending on caloric intake levels, based on age, sex, height, and weight. National data from 2003–2010 showed an increase in daily mean intake of total fruit among children and adolescents aged 2 to 18 years (4). However, large proportions of US children and adolescents do not meet the recommended daily servings of fruits and vegetables (2).

Although studies have examined fruit and vegetable consumption among children and adolescents (4–12), several gaps remain. Past research has used data from a single point in time or used combined data from 2007 through 2012 to conduct analyses (11,12). Other studies using trend analysis (4–10) reported mixed results or used data over a 2- to 3-year period (9,10), which may be too short to discern changes in consumption and or monitor patterns over time.

Far less research has investigated whether fruit and vegetable intake has changed over time among children and adolescents separately or whether those associations may vary by race and ethnicity. Some research suggests intake may vary among racial and ethnic subgroups in the US (4,6,8). One national study found nonsignificant increases in total fruit intake in 2009 versus 2003 among Mexican American, Black, and White children but a significant decrease in total vegetable intake among Black and Mexican American children aged 2 to 18 years (4). Moreover, a study that used data for 2009–2019 from a national adolescent survey reported a significant linear increase in daily fruit and vegetable intake among Black and White adolescents and a linear increase among Latino adolescents in fruit intake but no change in vegetable intake (8). Studies that investigate racial and ethnic variations are important, given that structural factors, including the social and built environments and related racism, often shape and constrain healthy food choices. Limited choices result from limited availability, access, and affordability of healthy food options in low income and racially segregated neighborhoods (13–15).

Studies that use more recent data from the past 10 years and diverse child and adolescent samples can help monitor and identify potential temporal changes in fruit and vegetable intake and inform future interventions. This is particularly important because many state and federal programs and policies in the US, such as the Healthy, Hunger-Free Kids Act (HHFKA) of 2010, were put in place to increase fruit and vegetable consumption among young people (16). The purpose of our study was to examine temporal changes in fruit and vegetable intake among California school-age children aged 5 to 11 years and adolescents aged 12 to 17 years, from 2011 through 2020, and to investigate whether any observed changes varied by race and ethnicity.

## Methods

### Data description

We used 2-year cross-sectional datasets from the child and adolescent samples collected in the California Health Interview Survey (CHIS) waves from 2011–2012 through 2019–2020 (17). Since 2011, 2-year survey cycles were used to collect CHIS data. The 2-year public-use data files can be accessed for research through application. We requested those files from CHIS because they contain more variables including detailed race and ethnicity variables. The largest state health survey in the nation, CHIS is a population-based telephone survey of household residents. Adolescents and children were randomly selected for interview from households participating in the survey. Adolescents were interviewed directly, and a knowledgeable adult caregiver completed the interview for children. CHIS includes large samples of racially and ethnically diverse populations. Survey data are collected in several languages, including English, Spanish, Mandarin, Cantonese, Vietnamese, and Korean. Data were weighted to account for the complex sample design and to adjust for nonresponse and for households without telephones. The samples consisted of 16,125 children aged 5 to 11 years and 9,672 adolescents aged 12 to 17 years. 

The CHIS sample design and methodology have been documented extensively elsewhere (18). Because these data are secondary and publicly available, our study was exempted from institutional board review by the authors’ academic institutions.

### Fruit and vegetable intake

Fruit and vegetable consumption was used as a dichotomous outcome variable and based on self-reported intake of 5 or more servings of fruits and vegetables a day. This variable was generated from 2 survey questions: “Yesterday, how many servings of fruits did you eat?” and “Yesterday, how many servings of vegetables did you eat?” CHIS constructed a “5+ Fruits/Vegetables a Day” variable that classified participants as having consumed 1) 5 or more servings of fruits and vegetables a day or 2) fewer than 5 servings of fruits and vegetables a day. For the adolescent sample, the constructed fruit and vegetable intake variable was available in the public-use data set across survey waves. Because the child data set for the 2013–2014 wave did not have the 5+ Fruits/Vegetables a Day variable but did have the variables derived from the 2 survey questions above, we constructed a dichotomous variable by using the same methods CHIS used for the adolescent sample (19).

### Temporal patterns

To examine temporal changes in fruit and vegetable consumption, we created a categorical variable to denote 3 different time periods: 2011–2012, 2013–2016, and 2017–2020. We chose these time periods because federal policies for school meals required more servings of fruits and vegetables in school meals beginning with the 2012–2013 school year, and some school meal requirements were relaxed in 2018 (20). We used 2011–2012 as the reference year.

### Covariates

Other covariates included self-reported race and ethnicity, generated by CHIS based on the California Department of Finance 2001 definitions (19) and classified as Latino, Non-Latino American Indian or Alaska Native, Non-Latino Asian, Non-Latino African American, Non-Latino White, Non-Latino Pacific Islander/Other 1 race, and Non-Latino 2 or more races — categories used in the CHIS survey. We combined American Indian or Alaska Native with Pacific Islander/Other 1 race into a category labeled as “Other 1 race.” We included self-reported age (centered at its mean); gender; annual household income as a percentage of the federal poverty level (FPL) classified as 200% FPL and above, 100% to 199% FPL, and 0% to 99% FPL; and educational attainment of the adult respondent whose household had an adolescent or child selected to take the survey, classified by CHIS as Bachelor’s degree or higher, Associate degree or some college or vocational school, 12th grade education/high school diploma, or less than a 12^th^ grade education; and the child’s citizenship status categorized as US-born citizen, naturalized citizen, or noncitizen.

### Statistical analyses

We estimated the distributions of the population characteristics: age, gender, race and ethnicity, poverty levels, education level of the adult respondent, and citizenship status. Distributions were estimated overall and for each time period (2011–2012, 2013–2016, and 2017–2020) and for children and adolescents separately. Statistical tests were used to compare the distribution of characteristics across years.

Modified Poisson regression models with robust standard errors were fitted to estimate the relative likelihood of reporting intake of 5 or more servings of fruits and vegetables in 2013–2016 and 2017–2020 compared with 2011–2012 by using the overall sample. We used Poisson instead of logistic regression as recommended, given that the outcome (intake of 5 or more servings of fruits and vegetables) was not rare, which results in overestimation of relative risk with logistic regression models (21). The Poisson regression models yield prevalence ratios (PR) rather than odds ratios. We adjusted the models for age, race and ethnicity, gender, family income, education level of adult respondents, and citizenship status. To investigate whether changes in daily fruit and vegetable intake varied significantly by race and ethnicity, a cross-product term between time period and race and ethnicity was added to the adjusted models. All analyses were conducted separately for children and adolescents and accounted for the complex sample design. Because we pooled the data across several 2-year cycles, we used published methods to generate new weights as recommended by researchers from the University of California, Los Angeles (22). A *P *value of <.05 was considered significant, including for tests of statistical interaction. We used Stata SE version 17 (StataCorp LLC) for our analyses, which we completed between July 2022 and September 2024.

## Results

Slightly less than a third (29.3%) of children and only 25.9% of adolescents in our samples reported consuming 5 or more servings of fruits and vegetables yesterday (Table 1). The age, gender, and racial and ethnic distributions were similar across time periods, except for 2017–2020, which had a smaller proportion of Latino children and a higher proportion of White children and adolescents, though the χ^2^ test was not significant (*P* =. 23, children and *P* =. 19, adolescents). Household income in the 2011–2012 and 2013–2016 periods were similar for children and adolescents, though the income distributions varied significantly across time (*P* =. 01), with greater proportions of higher income households in 2017–2020. Only among children did the education level of adult respondents vary significantly across time (*P* =. 001), where a greater proportion of household adults held a Bachelor's degree or higher in 2017–2020. Most children (94.5%) and adolescents (88.9%) were US-born citizens.

**Table 1:** Characteristics of Children and Adolescent Samples in California Health Interview Survey, Waves 2011–2020, Classified into 3 Time Periods^a^^,^ b

Characteristic	Children aged 5 to 11 years					Adolescents aged 12 to 17 years				
	Overall, n = 16,125	2011–12, n = 4,527	2013–16, n=6,227	2017–20, n=5,371	*P *value^c^	Overall, n = 9,672	2011–12, n = 2,799	2013–16, n = 3,847	2017–20, n = 3,026	*P *value^c^
**Consumed ≥5 fruits and vegetables a day, %**	29.3	25.2	31.7	28.8	.01	25.9	25.8	24.0	27.7	.23
**Age, mean (95% CI)**	8.1 (8.02–8.19)	8.1 (7.99–8.12)	8.1 (7.99–8.14)	8.2 (7.96–8.37)	.62	14.5 (14.50–14.58)	14.6 (14.54–14.63)	14.5 (14.44–14.59)	14.6 (14.48–14.61)	.25
**Gender, %**										
**Female**	49.2	49.7	48.7	49.5	.35	48.8	48.8	48.9	48.7	.83
**Race or ethnicity, %**										
African American	5.7	6.5	5.4	5.8	.23	5.3	4.9	5.6	5.2	.19
Asian	11.4	11.1	11.0	12.1		11.3	12.0	10.6	11.6	
Latino	49.3	52.7	51.1	45.4		47.5	47.0	49.0	46.2	
White	28.3	25.3	26.9	31.3		30.9	31.2	29.7	32.0	
2 or more races	4.3	3.9	4.6	4.1		4.1	4.0	4.5	3.7	
Other 1 race	1.0	0.5	1.0	1.3		1.0	0.9	0.7	1.3	
**Annual household income as percentage of federal poverty level (FPL), %**										
≥200 % FPL	55.3	51.4	51.5	61.4	.01	57.9	55.2	53.2	64.1	.01
100–199% FPL	21.7	23.7	23.0	19.4		22.2	23.3	23.9	19.8	
0–99% FPL	23.0	24.9	25.5	19.2		19.9	21.5	22.9	16.1	
**Adult educational attainment^d^, %**										
Bachelor’s degree or higher	39.2	35.1	36.6	44.1	.001	39.6	37.1	37.2	43.3	.25
Associate degree or some college or vocational school	21.7	24.9	23.3	18.4		21.9	23.1	23.8	19.4	
High school diploma	20.7	19.4	20.6	21.6		19.1	17.8	19.0	19.9	
Less than 12th grade	18.4	20.7	19.5	16.0		19.3	22.0	20.0	17.3	
**Citizenship status of child or adolescent, %**										
US-born citizen	94.5	94.0	94.3	94.9	.80	88.9	86.7	88.4	90.6	.21
Naturalized citizen or noncitizen	5.5	6.0	5.7	5.1		11.1	13.4	11.6	9.4	

a Based on combined 2-year data files from the California Health Interview Survey, waves 2011–2012, 2013–2016, and 2019–2020 (17).

b Weighted estimates.

c Based on χ^2^ test for categorical variables or ANOVA for continuous variables.

d Educational attainment of adult respondents whose household had an adolescent or child selected to take the survey.

Among children, results from regression models without adjustment for covariates showed that the prevalence of intake of 5 or more servings of fruits and vegetables was significantly higher in 2013–2016 (PR, 1.26; 95% CI, 1.11–1.43) and 2017–2020 (PR, 1.15; 95% CI, 1.01–1.30) compared with 2011–2012 (Table 2). After controlling for age, race and ethnicity, gender, socioeconomic factors (poverty level and education level of adult respondents), and citizenship status, the prevalence of consuming 5 or more servings of fruits and vegetables remained higher in 2013–2016 (PR, 1.25; 95% CI, 1.11–1.42) and 2017–2020 (PR, 1.13; 95% CI, 0.99–1.30) compared with 2011–2012. However, the prevalence ratio for 2017–2020 was no longer significant after adjustments.

**Table 2:** Prevalence Ratios Comparing Consumption of 5 or More Servings of Fruits and Vegetables a Day in 2013–2016 and 2017–2020 Relative to 2011–2012 Among Children and Adolescents, California Health Interview Survey^a^

Time-period	Children	Adolescents
**Unadjusted^b^**		
2011–12	Reference	Reference
2013–16	1.26 (1.11–1.43)	0.93 (0.79–1.11)
2017–2020	1.15 (1.01–1.30)	1.08 (0.90–1.29)
**Adjusted^b^**		
2011–12	Reference	Reference
2013–16	1.25 (1.11–1.42)	0.95 (0.80–1.12)
2017–2020	1.13 (0.99–1.30)	1.07 (0.91–1.27)

a Data source: 2-year data files from the California Health Interview Survey, waves 2011–2012 through 2019–2020 (17). Values are prevalence ratios (95% CI).

b Based on weighted Poisson regression models unadjusted or adjusted for age, gender, race and ethnicity, annual household income, adult educational attainment, and citizenship of child.

Among adolescents, we observed no evidence of changes in fruit and vegetable intake over time (Table 2). In the unadjusted model, the prevalence of intake of 5 or more servings of fruits and vegetables was somewhat lower in 2013–2016 (PR, 0.93; 95% CI, 0.79-1.11) and slightly higher in 2017–2020 (PR, 1.08; 95% CI, 0.90–1.29) compared with 2011–2012; however, none of these results were significant. After adjustment for covariates, the findings remained largely the same as those in the unadjusted model. 

We observed no evidence of differential temporal patterns in fruit and vegetable intake by race and ethnicity, either for children (*P* =. 93) or adolescents (*P* =. 47) (Table 3). The unadjusted and adjusted findings among children by race and ethnicity were generally similar to those reported for the child overall samples in Table 2. Among children in each racial and ethnic group, we observed greater reported intake of 5 or more servings of fruits and vegetables in 2013–2016 and 2017–2020 compared with 2011–2012, though the results were only significant for White and Latino children in 2013–2016 compared with 2011–2012. Among adolescents, there was no evidence of temporal changes in intake in any of the racial and ethnic groups in the unadjusted and adjusted analyses. Among White and Latino adolescents, intake of 5 or more servings of fruits and vegetables was higher in 2013–2016 and 2017–2020 compared with 2011–2012; however, these differences were not significant.

**Table 3:** Prevalence Ratios Comparing Consumption of 5 or More Servings of Fruits and Vegetables a Day Among Children and Adolescents in 2013–2016 and 2017–2020 Relative to 2011–2012, Stratified by Race and Ethnicity, California Health Interview Survey^a^

Time-period	White	Latino	Asian	African American
**Children^b^^,^ c**				
Unadjusted				
2011–12	Reference			
2013–16	1.28 (1.06–1.55)	1.22 (1.01–1.47)	1.51 (0.87–2.61)	1.24 (0.64–2.40)
2017–20	1.21 (0.97–1.52)	1.09 (0.75–1.61)	1.18 (0.47–2.99)	1.17 (0.63–2.15)
Adjusted				
2011–12	Reference			
2013–16	1.29 (1.06–1.56)	1.21 (1.00–1.46)	1.53 (0.89–2.64)	1.33 (0.71–2.50)
2017–20	1.23 (0.99–1.53)	1.08 (0.73–1.61)	1.19 (0.50–2.82)	1.26 (0.69–2.30)
**Adolescents^d^**				
Unadjusted				
2011–12	Reference			
2013–16	1.04 (0.84–1.29)	1.09 (0.82–1.45)	0.69 (0.41–1.16)	0.44 (0.19–1.04)
2017–20	1.14 (0.79–1.65)	1.22 (0.80–1.85)	0.81 (0.40–1.64)	0.70 (0.33–1.49)
Adjusted				
2011–12	Reference			
2013–16	1.06 (0.85–1.32)	1.11 (0.84–1.47)	0.69 (0.42–1.16)	0.47 (0.19–1.13)
2017–20	1.10 (0.75–1.61)	1.25 (0.84–1.87)	0.81 (0.41–1.61)	0.73 (0.31–1.74)

a Data source: 2-year data files from the California Health Interview Survey, waves 2011–2012 through 2019–2020 (17). Values are prevalence ratios (95% CI).

b Based on weighted Poisson regression models stratified by race and ethnicity, unadjusted or adjusted for age, gender, annual household income, adult educational attainment, and citizenship of child.

c *P* value for interaction =. 93. Calculated by Wald test.

d *P* value for interaction =. 47. Calculated by Wald test.

## Discussion

Ours is one of the first studies to investigate temporal patterns in the consumption of fruits and vegetables by using recent data from the largest state health survey (CHIS) and comparing 3 time periods (2011–2012, 2013–2016, and 2017–2020) to investigate potential racial and ethnic differences in temporal patterns of intake in the most populous and diverse state in the nation (23). We found that in California, children’s consumption of 5 or more servings of fruits and vegetables increased in 2013–2016 and 2017–2020 compared with 2011–2012, though the increase in 2017–2020 was smaller and not significant after controlling for sociodemographic factors. These temporal changes in the amounts of fruits and vegetables consumed were not evident among adolescents. We saw no clear evidence of differential temporal changes in fruit and vegetable intake by race and ethnicity among children or adolescents.

The greater likelihood of reporting intake of 5 or more servings of fruits and vegetables a day among children that we report here is consistent with previous research examining changes from 2015 to 2017 (9). Our study adds to that evidence by investigating a longer time period (2011–2020). Prior US research in children examined fruit or vegetable intake separately and reported an increase in fruit consumption in a time period before 2011, making it difficult to directly compare findings with our study results (4).

Our study did not observe evidence of temporal changes in reported fruit and vegetable consumption among California adolescents, in contrast to prior studies that found an increasing trend in fruit and vegetable intake (5,9). Differences between these studies and our study’s findings may be due to differences in the time periods in which the data were collected or the ways in which intake of fruits and vegetables was measured. For example, one study used a shorter time period, from 2015 through 2017 (9), whereas another study used the Youth Risk Behavior Surveillance System, which measured intake in terms of the number of times fruits and vegetables were consumed in the past 7 days (5). Other studies involving adolescent samples examined fruits or vegetables separately (4,6–8,10), precluding our ability to directly compare our results against those studies.

The reasons for the elevated prevalence in fruit and vegetable intake we observed among children in 2013–2016 and the lack of evidence of temporal changes in adolescents may be multifactorial, with complex interplays between individual, sociocultural, policy, or environmental factors. Though age, gender, and socioeconomic status have been associated with children’s fruit and vegetable intake (24), we controlled for them in the analyses; thus, they are unlikely to explain the findings we reported among children. Psychosocial factors such as food preferences may also influence intake, yet the CHIS data set did not have these indicators, restricting our ability to include them in our analyses. Evidence indicates that access to fruits and vegetables in the home and parental support are associated with fruit and vegetable consumption among children (25), but we did not have home food environment data for our study. Research is needed to examine temporal changes in fruit and vegetable intake at home and other food venues, including schools.

Many policies have been enacted to improve diet quality among children in schools. For example, starting in the 2012–2013 school year, HHFKA required schools to increase fruit and vegetable servings in the lunch program (16). Many, but not all, studies found this policy to be associated with improved fruit and vegetable intake (26). A study of trends in diet quality among children and adolescents aged 5 to 19 years found foods in schools improved after 2010 because of increases in whole grains and fruits and vegetables, a timing that coincides with the HHFKA 2010 policy (27). It is plausible that the higher prevalence of fruit and vegetable intake we observed among children in 2013–2016 may be partly due to the implementation of HHFKA. The prevalence ratio among children remained higher but was weaker and nonsignificant in 2017–2020 compared with 2011–2012. This weaker association may have been related to the increased flexibilities in the implementation of HHFKA enacted in 2018 (20). However, other factors also may have played a role, including school closures because of the COVID-19 pandemic, which severely limited access to school meals.

The food environments in residential neighborhoods and near schools, including convenience stores and fast-food restaurants, are unlikely to explain the observed temporal increase in fruit and vegetable intake among children. Prior research showed that these outlets tend to offer unhealthy food and beverages high in sodium, fats, and sugars (28), and their proximity to schools is associated with the consumption of snacks, desserts, fast food, and sugary beverages (29,30). In contrast, adolescents may be more likely to interact with food environments near schools because adolescents have greater mobility, access to pocket money, and are less likely to participate in school meals. If adolescents substitute unhealthy food and beverages obtained from food outlets near schools or their homes for fruit and vegetable intake, this could, in part, explain the absence of changes in intake in this population. It remains unclear whether food outlets near schools increased offerings of fruits and vegetables over the time period covered in our study. Food stores and restaurants are 2 major sources of calories for children and adolescents (27). Thus, future research is needed to monitor the extent of food offerings, including fruit and vegetables, in outlets near schools and residential locations and whether changes over time contribute to consumption of healthy and unhealthy foods.

We found no evidence of differential temporal patterns in fruit and vegetable consumption by race and ethnicity among children or adolescents. To the best of our knowledge, our study is the first to examine whether temporal patterns in intake of 5 or more servings of fruits and vegetables vary across race and ethnicity among children and adolescents separately. In contrast to our study findings, a national study observed significant increases in fruit and vegetable consumption from 2009 through 2019 among Black and White adolescents, whereas Hispanic adolescents saw a significant increase only in fruit intake and no changes in vegetable consumption (8). Based on a sample of children and adolescents combined, a national study examined fruit and vegetable intake separately and reported no changes in fruit intake from 2003 through 2010 among racial and ethnic groups. However, vegetable intake declined among Mexican-American and Non-Hispanic Black participants, whereas Non-Hispanic White participants saw no changes in the same time period (4). Our study conducted its analyses separately for children and adolescents, whereas some of the previous studies examined adolescents only (8) or combined samples of children and adolescents (4), which may partly explain the differences in findings between those studies and our study.

### Implications

The data in this study are repeated cross-sectional surveys of the population, which enabled us to estimate population changes but not child-level longitudinal changes in consumption. Population changes remain important for understanding public health implications of fruit and vegetable intake. Although our findings suggest temporal improvements in intake of 5 or more servings of fruits and vegetables a day among children in 2013–2016, the weaker evidence of changes in the later time period (2017–2020) suggests that changes may be nonlinear or that other factors may have played a role. Future studies are needed to evaluate changes in fruit and vegetable intake, comparing the period when HHFKA 2010 was implemented, as well as before and after the COVID-19 pandemic. It is important to note that approximately 70% of children and adolescents in the data we used reported consuming fewer than 5 servings of fruits and vegetable a day across time periods, reflecting a significant challenge for planning future public health nutrition policy and related interventions. Given previous research that found that participation in school meals programs is associated with improvements in diet quality among children (31), strengthening existing nutrition programs and policies, such as the Community Eligibility Provision, Universal School Meals, and the Fresh Fruits and Vegetables programs (32), remains critical. These programs can increase children’s access to enough fruits and vegetables in schools (33), settings where they spend a large proportion of their waking hours and may contribute to improved health outcomes in later life, including prevention of obesity (34). Existing longitudinal studies of fruit and vegetable intake are limited (35,36); thus, additional research is needed to evaluate and monitor consumption patterns. Future studies are also needed to examine associations between changes in fruit and vegetable intake and changes in health outcomes, including body weight and chronic diseases.

### Limitations

Our study had several limitations related to the measurement of fruit and vegetable intake, the cross-sectional nature of the data, and small sample sizes among racial and ethnic subgroups. Self-reported measures of fruit and vegetable intake versus observed measures tend to underreport energy intake, and parents responding for their children may be influenced by social desirability (37). The self-reported measure of intake of fruits and vegetables was based on the day before the survey, potentially raising recall bias, although this methodology has been widely used previously. Although our study’s data were cross-sectional, the availability of these data enabled us to examine changes in fruit and vegetable intake over time. The lack of a significant statistical interaction may be due to small sample sizes among racial and ethnic subgroups, especially in the adolescent sample. Given the focus on California’s child and adolescent populations, this study’s findings may not be generalizable to children and adolescents in other US states.

### Conclusion

We observed greater self-reported consumption of 5 or more servings of fruits and vegetables among children in California in the 2013–2016 period and to a lesser degree in 2017–2020, compared with 2011–2012. We observed no clear patterns among adolescents. We found no evidence of temporal variations in intake by race or ethnicity in children or adolescents. The observed greater intake in fruit and vegetable consumption in 2013–2016 versus 2011–2012 among children is encouraging, although a very high proportion of children and adolescents do not consume 5 or more servings of fruits and vegetables a day. Continued efforts are needed, especially local, state, and federal policy interventions, to increase child and adolescent intake of fruits and vegetables at the population level.
